# Education Research: Introduction of a Standardized Communication Card to Facilitate Patient-as-Teacher Training for Medical Students in the Neurology Clerkship

**DOI:** 10.1212/NE9.0000000000200115

**Published:** 2024-03-15

**Authors:** Carmen Priego-Pérez, Punthitra Arpornsuksant, Rachel Marie E. Salas, Charlene E. Gamaldo, Monica Lemmon, Roy E. Strowd, Doris G. Leung

**Affiliations:** From the Facultad de Medicina (C.P.-P.), Universidad de Córdoba, Spain; Department of Neurology (P.A., R.M.E.S., C.E.G., D.G.L.), Johns Hopkins Medicine, Baltimore, MD; Department of Pediatrics and Population Health Sciences (M.L.), Duke University School of Medicine, Durham; Department of Neurology (R.E.S.), Wake Forest University School of Medicine, Winston-Salem, NC; and Kennedy Krieger Institute (D.G.L.), Baltimore, MD.

## Abstract

**Background and Objectives:**

Enhanced communication has been recognized as an effective strategy to improve patient safety and care quality. While some communication skills can be taught in traditional didactic settings, learning from patient encounters is critical. Currently, patients are becoming increasingly involved as teachers for medical students within both the clinical and classroom setting. The goal of this study was to characterize medical student reflections about patient interactions using a standardized Patient and Teacher Communication Card. We aimed to identify how the introduction of this tool changed students' follow-up practices and affected patient care.

**Methods:**

We used a cross-sectional concurrent mixed-methods study to characterize student-patient communication. Medical students taking the neurology clerkship between 2017 and 2022 were asked to complete the Communication Card during at least 1 patient encounter. The Communication Card was used to generate a learning opportunity by providing questions for the students to ask the patient. Following the encounter, the card collected qualitative data from the student's perspective through 2 open-ended questions: (Q1) How has the card changed how you follow-up with patients after rounds? (Q2) How did this follow-up affect patient care? We used a conventional content analysis approach to characterize student responses.

**Results:**

A total of 460 students completed the card (MS2: n = 67 [14.6%]; MS3: n = 260 [56.5%], and MS4: n = 133 [28.9%]). Students cited 4 ways in which the card changed their follow-up with patients: (1) ensuring understanding; (2) following up more; (3) building rapport; and (4) guiding challenging conversations. Ensuring understanding was cited by half of the students in all years. Students cited ways in which the card affected patient care: (1) prompting further discussion with the team and/or patient; (2) impression of the patient feeling more comfortable; (3) addressing patient concerns; and (4) impression of increased trust.

**Discussion:**

Overall, students' reflections after patient conversations were very positive. Future work should consider studying the impact of this communication tool on patients' perspectives and determine whether they align with the student's perception. In addition, implementation of a Communication Card throughout the other clerkships should be considered to enhance the medical school curriculum.

## Introduction

Effective communication is one of the most important contributors to patient care quality and is the basis of the physician-patient relationship.^1,2^ However, despite being one of the core competencies for the American Council for Graduate Medical Education, there is no standardized curriculum for obtaining these communication skills during medical school.^2^ Within the field of neurology, previous research indicates that there may be significant gaps in the development of communication skills at the medical school level. Although neurology care teams frequently need to facilitate challenging conversations with patients and families (including discussions about poor neurologic prognosis, prognostic uncertainty, new disability, terminal diagnoses, end of life, or other topics), one study found that at the end of the neurology clerkship, 87% of students desired additional communication skills training during the clerkship itself.^1,3^

One potential approach for improving communication skills among medical trainees has been to incorporate patients into the teaching process. Patients have always been an integral part of medical education, but their involvement tended to be passive until the 1960s.^4^ Within the medical education system, patients (including standardized patients) have increasingly become involved with medical trainees as their teachers. A prior systematic review found that patients' main roles in medical education include teaching communication skills to trainees, especially through standardized patients or within the classroom setting.^5^ Communication skills training via standardized patients and workshops has also been tested across some institutions with promising results on Objective Structured Clinical Examinations and breaking bad news assessment tools.^6-9^ The benefits of using patients-as-teachers for students include a perceived relevance of learning, increased learner satisfaction, communication skills, reduced anxiety, improvement in acquisition of skills, increase in respect for patients, and placing learning in context.^10,11^ Furthermore, benefits for patients include findings ways to use their disease, knowledge, and experience positively, acknowledging their expertise, creating a sense of empowerment, learning about their condition, becoming familiar with training doctors, and feeling useful in providing practical help for students.^10^

In the clinical setting, one widely used method for patient-as-teacher communication is the Teach-Back method, which involves asking patients to explain what they learned or understand about their condition or treatment. This helps ensure clarity, assess comprehension, and reinforce learning for the patient. To incorporate this technique into the neurology core clerkship at our institution, we introduced a Patient-as-Teacher Communication Card to guide conversations between students and patients about the patient's understanding of their medical condition and treatment.^11^ The Communication Card provided 3 questions for students to ask patients on their rotation and asked the student to reflect on their experiences after using those questions. We hoped these questions would enhance the role of the medical student as an intermediary between patients and the medical team by providing opportunities to gain new insight on the patient's perspective, elicit valuable information that could be brought back to the team, advocate for their patients, and improve the patient's experience and quality of care. The cards were initially developed as a communication skills educational intervention. In our prior studies, we found that crucial conversations occur frequently in the neurology clerkship and there was an opportunity to enhance debriefing.^1^ We identified that students were frequently having these discussions with patients and benefited from debriefing with the team. The card was created to prompt a debrief with the resident and physician team after such a conversation. In piloting it, we found that the card allowed patients to contribute to teaching and debriefing of students and the questions on the card were modified to its final form.

The primary aim of this study was to characterize the impact of a Patient-as-Teacher Communication Card on communication skills among students. We hypothesized that most students would appreciate having a standard set of structured questions to spark conversation with the patient and initiate further discussion with the medical team, possibly leading to further changes to care. Second, we aimed to determine whether there were differences in responses between students in different years of training.

## Methods

We used a cross-sectional concurrent mixed-methods study. Open-ended responses were analyzed using a conventional content analysis approach.^12^ All medical students at Johns Hopkins School of Medicine who enrolled in the neurology clerkship between January 2017 and February 2022 completed the card and were consecutively selected. Notably, no data were collected between March 2020 and June 2020 due to the coronavirus disease 2019 (COVID-19) pandemic and halting of in-person clerkship activities. Between June 2020 and July 2021, the neurology core clerkship was modified to be 3 weeks long due to the pandemic. Before March 2020 and after July 2021, the clerkship was 4 weeks long.

Before March 2020, the clerkship included 2 weeks of inpatient at an academic institution, 1 week of clinic, and 1 week at a community hospital. Since June 2020, the clerkship involved a single site of either inpatient wards or outpatient clinical settings. Students on an inpatient service were assigned to a rotation in either stroke neurology, general neurology, pediatric neurology, or neurology consults. Students at an outpatient site were placed in various general adult and pediatric neurology clinics or specialized clinics including epilepsy, neuromuscular, neuro-oncology, movement disorders, sleep medicine, and neuroimmunology.

### The Communication Card

At the start of the neurology clerkship, students were each given a Communication Card. Each student was required to use the card with at least 1 patient after morning rounds during the clerkship. Completion of the assignment was required to pass the clerkship. Medical students were instructed to check in with patients after rounds or after complex and difficult discussions, using the Communication Card to help guide the conversation. It was up to the student's discretion whether to introduce the card itself to patients and families. Students either memorized the questions before entering the room or had the card in the room with them. The card included 3 questions to ask the patient: (1) What was discussed by your medical team? (2) Were all your questions addressed? (3) What remaining questions do you have? (Figure). The goal of the questions was for the patient to be the student's teacher and help the medical student to understand the patient's perspective, advocate for the patient, and improve the patient's experience. After speaking with the patient using the Communication Card, students were instructed to discuss what they found with other health care team members as needed.

**Figure:**
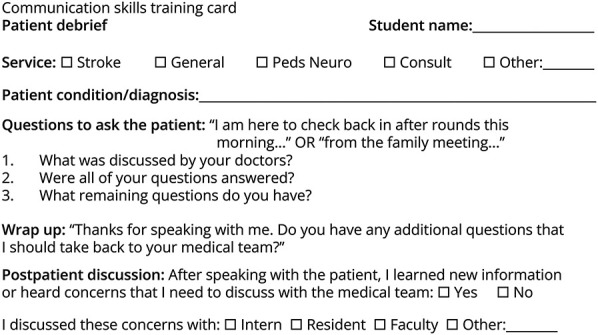
Communication Skills Training Card The communication skills training card that students completed during the neurology core clerkship, with (A) student name, clerkship service, and outline of the conversations with questions to ask and post-patient discussion. (B) Debrief questions for the student to complete after speaking to the patient.

After speaking with the patient and the health care team, students had to document the following: (1) the service where they were rotating, (2) the patient's condition, (3) whether they had a postpatient discussion with the medical team, (4) which members of the team they brought up patient concerns or new information with, (5) number of patients they used the card with, and (6) which members of the team the student debriefed their conversation with.

Finally, students were asked to write their reflections on the card for 2 open-ended questions: (Q1) How has the card changed how you follow-up with patients after rounds? (Q2) How did this follow-up affect patient care?

### Analyses

We used a mixed-methods approach: The 2 open-ended responses were analyzed using conventional content analysis, using NVivo qualitative software (version 12). Two researchers (C.P.-P. and P.A.) jointly created a codebook to characterize student responses through an initial review of the data. The codebook was derived from the data itself through this initial review, rather than from an existing theory or prior research.^13^ We then independently applied the codebook to the responses, and 15% of responses were coded by both coders to ensure adequate interrater reliability. Interrater reliability was assessed with Cohen κ (κ was >0.8 for all). Each response had at least 1 code. Each response to the 2 open-ended questions was classified into 1 or more category. There were initially 5 main response codes for question 1 and 4 codes for question 2. Both questions also included codes for “not answered,” “no change,” and “other.” Through an iterative process, 2 response codes for question 1 were combined under 1 code, resulting in 4 main response codes per question. Students were separated into 3 tally groups: second-year students, third-year students, and fourth-year students. Descriptive statistics were used to define student demographics and track clerkship sites.

### Standard Protocol Approvals, Registrations, and Patient Consents

All data were secured and saved in Johns Hopkins Safe Desktop. This study was reviewed and exempted from convened review by the Johns Hopkins Medicine Institutional Review Board.

### Data Availability

Anonymized data will be shared by request of qualified investigators.

## Results

A total of 460 medical students were enrolled in the neurology clerkship and completed the communication card. All student responses were included in the study. Respondents included students in the second (n = 67, 14.6%), third (n = 260, 56.5%), and fourth (n = 133, 28.9%) years of medical school. The average age was 26.0 years (SD = 2.6 years) (Table 1). Quotes are included to represent and illustrate examples of student responses for each code.

**Table 1:** Demographics and Clerkship Tracking in the Neurology Clerkship

Demographics	Values (total n = 460)
Age, y, mean (SD)	26.0 (2.6)
Medical school years, n (%)	
2	67 (14.6)
3	260 (56.5)
4	133 (28.9)
Clerkship site/site, n (%)	
General inpatient	144 (31.3)
Consult	142 (30.9)
Pediatric neurology	73 (15.9)
Stroke	63 (13.7)
Neurocritical care unit	20 (4.3)
Outpatient	15 (3.3)
Not answered	3 (0.7)
Postpatient discussion, n (%)	354 (77)
No. of patients, n (%)	
1	130 (28.3)
2–4	283 (61.5)
5 or more	47 (10.2)

### Question 1: How Has the Card Changed How You Follow-Up With Patients After Rounds?

Students shared that the communication card affected their follow-up with patients in rounds in 4 key ways (Table 2): (1) ensure patient understanding, (2) follow-up more with patients, (3) build rapport with patients, and (4) guide challenging conversations (Tables 2 and 4). A few students either did not answer the question (0.4%, n = 2), reported no change (10.9%, n = 50), or reported a result that did not fit into the other categories (6.1%, n = 28).

**Table 2:** Categories for Responses to Question 1 and Examples of Each

Q1. How has the card changed how you follow-up with patients after rounds?	
Category (n, %)	Quotes
Helped me guide challenging conversations (N = 154, 33.5%)	“It provided me with a script to approach what can be an intimidating process.” —Third-year student 10 “This card gives me a framework to model my interactions with patients. I have some structure for these difficult conversations.” —Second-year student 4
Ensure patient's understanding (N = 254, 55.2%)	“If a patient was given a confusing diagnosis or had lots of test/results to understand, I would make more of an effort to check in to see if they understood or had more questions.” —Third-year student 11 “This card helped me as a reminder to always be sure that the patient has follow-up with the whole team for every concern or health question.” —Third-year student 12
Build rapport with patients (N = 186, 40.4%)	“It helped me to be more engaged with my patient's care. I felt it built a stronger rapport between the patient and me.” —Second-year student 5 “The patients were extremely grateful for the extra attention; I likely would not have initiated this conversation without the card.” —Third-year student 13
Follow-up and spend more time with patients (N = 219, 47.6%)	“I realized sometimes patients/family take more time to process information, and will often have more questions later in the day, so it would be important to follow-up.” —Fourth-year student 5 “The approach outlined in this communication card gave me more time to focus on one-to-one communication with patients and to explore their questions/concerns more thoroughly. The team was often very time constrained during rounds so I felt as if this card allowed me to maximize the efficacy of the extra time I was able to spend with patients.” —Third-year student 14
No change (N = 50, 10.9%)	“I have always tried to do a second follow-up with my patients as I feel it helps build rapport more than anything. Although it did not change my follow-up, it reinforced that this is an important thing to do.” —Fourth-year student 6
Other (N = 28, 6.1%)	“I learned that the patient wasn't an entirely reliable historian, and it encouraged me to reach out to the patient's family for more detail.” —Fourth-year student 7 “Able to seek more instant feedback after changes to the medical plan.” —Fourth-year student 8
Not answered (N = 2, 0.4%)	The question was left blank

**Table 3:** Categories for Responses to Question 2 and Examples of Each

Q2. How did this follow-up affect patient care?	
Category (n, %)	Quotes
Further discussion with the medical team was had (N = 160, 34.8%)	“I was able to bring to the team concerns of the patient and family that hadn't been mentioned on rounds and strengthen the relationship between the patient and team. This helped the patient feel more comfortable in expressing their concerns and contribute additional family and social history that could help us in their management course.“ —Fourth-year student 10 “I think that our private discussion benefited building upon the shared decision making with the team. I believe my patient felt cared for knowing I was on his team and expressed my concerns for his non-medical needs.” —Third-year student 15
Students had the impression that the patient felt more comfortable (N = 159, 34.6%)	“It allowed the patient to feel more at ease with his diagnosis and next steps in care.” —Third-year student 16 “Gave the patient and daughter a better understanding of the situation and plan going forward, helped to reduce existing anxiety.” —Third-year student 17
Patient's concerns were addressed by the medical team (N = 153, 33.3%)	“I was able to identify specific concerns to bring back to the fellow and attending that helped each patient's specific needs yet addressed efficiently.“ —Fourth-year student 11 “After following up with this patient after rounds I learned more about his mood, including the loneliness he was experiencing in the hospital and his concerns about how the hospitalization would affect his recovery from addiction. I was able to put in a request for a chaplain and a peer recovery specialist to speak with him. I was also able to spend more time talking to him. He expressed appreciation for these interventions.“ —Second-year student 5
Students had the impression that the patient felt more heard and trusting of their medical team (N = 135, 29.3%)	“In regard to the patient's emotional response to their hemorrhagic stroke, while I couldn't predict how the patient would do in the long term, I hope that allowing them to express their emotions and making them feel heard was therapeutic.” —Third-year student 18 “It took more time to explain our thought process, increasing patient's family's confidence in our care.“ —Third-year student 19
Other (N = 52, 11.3%)	“In my patient's care, it gave another opportunity to reflect on their substance use. I think it may lead to positive behavior change in the future and added a humanistic component to the patient's care.” —Fourth-year student 12 “It kept the team up-to-date and provided terrific handoff as we ended up admitting the patient.” —Third-year student 20
No change (N = 21, 4.6%)	“I think in my case it did not affect management, because the patient had a great group in his care, but I can see how in the future it could be extremely helpful for keeping the patient in the loop.” —Third-year student 21
Not answered (N = 0, 0%)	The question was left blank

#### Helped Ensure Patient Understanding

Most students were able to answer questions from patients, increasing their understanding of their condition. This was especially helpful for patients who had an impaired neurologic function or who had received a new diagnosis and had a poor understanding of it. Students identified gaps in the conversation that took place during rounds, and this was useful to ensure that the team and the patient were on the same page.

It reinforced the importance of checking in with patients to ensure that they don't have lingering concerns that went unaddressed and that they understand the current plan for management. —Third-year student 1.

This communication card allowed me to assess how well the patient and/or their family understood what had been discussed during rounds. It also gave me guidance regarding how to elicit questions and concerns from the patient and their families so that necessary information could be provided to them and the team would have a better understanding of how the family was making sense of all that was occurring. —Third-year student 2.

Overall, most of the students cited the impact of the card on patient understanding (n = 54 [55.2%]). Students in their third and fourth years of medical school cited this finding more frequently (MS2: 28 [41.8%]; MS3: 152 [58.5%]; and MS4: 74 [55.6%]).

#### Follow-Up and Spend More Time With Patients

Students mentioned the importance of giving patients more time and space to process, especially after giving them a new diagnosis or poor prognosis. They described taking extra time to talk privately with patients after rounds because rounds can be rushed, crowded, intimidating, and overwhelming. Students reported that the card encouraged them to be more purposeful with their follow-ups, to check in with additional patients, and to spend more time during each follow-up. Consequently, students told that sometimes they identified new patient concerns and relayed that information to the team. They emphasized that spending more time with patients outside of rounds allowed them to talk with them in a more private setting without the rest of the team, and patients expressed concerns and asked questions that they were not comfortable asking in front of the whole team.

It showed me how important it is to repeatedly ask patients for any further questions they have and to give patients enough time to let information process and marinate, especially for new diagnoses. The patient was very tearful when we first told her she likely had Parkinson disease and she was with her husband and son. We spent a very long time with them and they were very appreciative of us for letting them exhaust all their questions and for doing a thorough exam. —Fourth-year student 1.

I initially thought I should only visit patients if I had a concrete, pertinent update for them. Through this card, I became more comfortable just going to their room to check up on them, answer their concerns, and sometimes glean new information. It definitely enriched my experience. —Third-year student 3.

Nearly half of students shared that the card enabled them to follow-up and spend more time with patients (n = 219 [47.6%] of students in total). This finding was cited more commonly by students later in their training (MS2: 22 [32.8%]; MS3: 127 [48.8%]; and MS4: 70 [52.6%]).

#### Built Additional Rapport With Patients

Some students mentioned that, after using the Communication Card, some patients felt free to express their worries, concerns, and feelings, instead of only discussing their symptoms. They described connecting with family members and being more aware of their wishes. Students stated that sometimes the communication card allowed them to respond to emotional needs of patients, which was helpful for the patients to feel more comfortable while in the hospital. They described some patients feeling lonely and that after using the communication card they spent more time accompanying them and putting them at ease. Students perceived an increase in patients' trust and satisfaction and that some patients expressed feeling heard and genuinely cared for. Some even said that the communication established them as the primary communicator or even a point person to relay additional concerns to the team.

It allowed me to follow-up on the patient's concerns and specific questions/sources of anxiety. It also facilitated the patient being able to express frustration and I believe this was helpful for him. —Second-year student 1.

I thought it was a useful reminder to follow-up with patients, even if they didn't necessarily seem to have questions during the AM rounds, they often had more questions in a 1-on-1 setting. I also think they appreciated people checking in on them throughout the day, particularly during this COVID-19 times as I think many felt lonely and simply enjoyed having someone to chat with. —Fourth-year student 2.

The concept of building rapport was cited more frequently by students later in training, although there were not statistically significant differences between years (MS2: n = 19 [28.4%]; MS3: n = 113 [43.5%]; and MS4: n = 54 [40.6%]).

#### Helped Me Guide Challenging Conversations

Students said that the Communication Card provided a great template to approach challenging conversations. Because the follow-up was more structured and focused, they felt less hesitant going back to see patients after rounds because they knew what questions they should ask.

Students said it helped them understand the implications of the words they say and encouraged them to use the correct words and avoid jargon. They reported it also gave them guidance regarding how to elicit questions and use an open-ended approach to interviews. Some students used the Teach-Back method during these conversations. Finally, they described that the card provided a framework that helped them feel more confident.

This card gave me a format with which to follow-up to make me more confident in addressing patient concerns and approaching these topics after difficult information was shared as they deal with the illness of a loved one. —Fourth-year student 3.

It made my follow-up more structured and focused—I felt less hesitant going back to see my patients after rounds because I knew what questions I should ask. —Third-year student 4.

Approximately a third of students found that the Communication Card helped provide a framework for them to guide challenging conversations (n = 154 [33.5%]) in all years (MS2: 22 [32.8%]; MS3: 93 [35.8%]; and MS4: 39 [29.3%]).

### Question 2: How Did This Follow-Up Affect Patient Care?

Codes identified for Q2 included the following: (1) prompting further discussion within the team and/or patient; (2) impression of patient feeling more comfortable; (3) addressing patient concerns; and (4) impression of increased trust. These were equally distributed, with approximately one-third of students citing each (Tables 3 and 5). A few students reported no change (4.6%, n = 21) or reported a change that did not fit into the other categories (11.3%, n = 52). There were no students who left this question unanswered.

**Table 4:** Comparison of Student Response Categories for Question 1 Between Medical Student Year

	All students (N = 460)	Second-year students (N = 67)	Third-year students (N = 260)	Fourth-year students (N = 133)
Build rapport with patients, n (%)	186 (40.4)	19 (28.4)	113 (43.5)	54 (40.6)
Ensure patient understanding, n (%)	254 (55.2)	28 (41.8)	152 (58.5)	74 (55.6)
Follow-up and spend more time with patients, n (%)	219 (47.6)	22 (32.8)	127 (48.8)	70 (52.6)
Helped guide difficult conversations, n (%)	154 (33.5)	22 (32.8)	93 (35.8)	39 (29.3)
No change, n (%)	50 (10.9)	4 (6.0)	29 (11.2)	17 (12.8)
Other, n (%)	28 (6.1)	8 (11.9)	11 (4.2)	9 (6.8)
Not answered, n (%)	2 (0.4)	1 (1.5)	1 (0.4)	0 (0.0)

**Table 5:** Comparison of Student Responses for Question 2 Between Medical Student Years

	All students (N = 460)	Second-year students (N = 67)	Third-year students (N = 260)	Fourth-year students (N = 133)
Students had the impression that the patient felt more comfortable, n (%)	159 (34.6)	20 (29.9)	95 (36.5)	44 (33.1)
Further discussion with the medical team, n (%)	160 (34.8)	23 (34.3)	96 (36.9)	41 (30.8)
Students had the impression that the patient felt more heard and trusting, n (%)	135 (29.3)	25 (37.3)	69 (26.5)	41 (30.8)
Patient's concerns were addressed, n (%)	153 (33.3)	31 (46.3)	83 (31.9)	39 (29.3)
Other, n (%)	52 (11.3)	7 (10.4)	26 (10.0)	19 (14.3)
No change, n (%)	21 (4.6)	3 (4.5)	13 (5.0)	5 (3.8)

#### Prompting Further Discussion

Discussion was about different topics such as new test results, patient's concerns, and additional information that the student had identified and fed back to the team. Students provided examples where these discussions led to a change in treatment, to a consideration of patient's preferences, to better continuity of care, to tailoring conversations, to strengthening of the relationship between the patient and team, and to clarification of a diagnosis.

Discussing additional needs with faculty lead to further discussion about the patient's mood, and a discussion with the patient about if he would be interested in seeing a therapist, which he agreed to, as well as agreeing to finally see a psychiatrist which had been suggested before. —Third-year student 5.

This improved the patient's care, they now have an additional avenue to relay their concerns to the team after rounding. It was also a nice reminder to the team regarding the patient's values and expectations. —Third-year student 6.

A third of the students (n = 160 [34.8%]) mentioned prompting further discussion with the team. There were minimal differences between school year (MS2: n = 23 [34.3%]; MS3: n = 96 [36.9%]; and MS4: n = 41 [30.8%]).

#### Impression of Patient Feeling More Comfortable

Students cited having the impression of patients feeling more at ease with their diagnoses and plan. They reported that in many cases, this was due to a better understanding of the diagnosis and next steps in their care. Some students mentioned that this led to a better adherence to treatment.

I was able to find out how anxious the patient was about a potential cancer diagnosis, given his history of cancer. He also expressed worries about being independent. This allowed our team to spend a bit more time each morning on rounds to reassure him. —Third-year student 7.

I believe this led to patients having a better understanding of their condition and risk factors (especially for stroke, patients with transient ischemic attack) and perhaps they will have a better adherence to their medication regimens/ lifestyle modifications. —Third-year student 8.

This code was cited by a third of students (n = 159 [34.6%]). It was equally distributed by students in all years (MS2: n = 20 [29.9%]; MS3: n = 95 [36.5%]; and MS4: n = 44 [33.1%]).

#### Addressing Patient's Concerns

Students gave examples of concerns they could address themselves or bring back to the team, such as discharge planning, prognosis of a new diagnosis, or social aspects of care.

I ensured that there was a good follow-up plan for her main concern about her ability to drive and double checked the Maryland state Department of Motor Vehicles requirements for returning to driving after a stroke. —Third-year student 9.

The patient's family was having difficulty understanding his condition and prognosis, and by following up, I was able to curate some questions that I brought to the residents' attention, which was addressed by when we saw them the next day. —Second-year student 2.

A third of students (n = 153 [33.3%]) mentioned addressing concerns from patients. Students early in training cited this code more frequently (MS2: n = 31 [46.3%]; MS3: n = 83 [31.9%]; and MS4: n = 39 [29.3%]).

#### Impression of Increased Trust

Students perceived an increase in trust after using the card. They described patients expressing appreciation for the extra attention and for feeling heard and cared for. They also described going out of their way to listen to patients' thoughts and concerns and the impression of providing better care because they were treating the patient more than the disease.

It helped the patient feel heard, understood and cared for. —Second-year student 3.

We were able to obtain up to date information to coordinate more efficient and effective care for my patients. In addition, the patient's families and caretakers were also able to build stronger trust and rapport with the team as a result. —Fourth-year student 4.

Like the other codes, this was cited by a third of students (n = 135 [29.3%]). It was equally distributed in all groups (MS2: n = 25 [37.3%]; MS3: n = 69 [26.5%]; and MS4: n = 41 [30.8%]).

## Discussion

During medical school, students should not only learn clinical skills but also be able to communicate effectively with patients and their families regarding their diagnoses and plans. Prior research has found that during a neurology clerkship, students are exposed to difficult conversations and would prefer additional clinical communication skills training.^1^ We introduced a structured communication training card during the neurology clerkship and characterized postconversation student reflections to bridge this gap. In this study, patients were used as teachers to help students understand their perspective and provide further insight into their care. The card provided a framework for the student to follow and prompted debriefing with the medical team afterward.

Overall, we found that most students appreciated the framework we provided when following up with patients. They describe increased patient understanding, follow-up, patient-team rapport, and ease of conversation guidance. While students learn clinical material on the wards, they do not always learn how to frame a patient conversation. Having a predetermined framework helped students feel more confident when approaching patients after rounds. We found that the most significant quantitative differences were that students later in training reported that following up helped patient understanding and building rapport. This suggests that students later in training feel more prepared to interact with patients and share their medical knowledge. Inversely, it also suggests that introducing the Communication Card early in training can be very beneficial to help medical students feel more comfortable following up with patients. Using a conversation framework during medical school can aid students in their future practice when approaching more challenging conversations, while reminding them to avoid jargon by asking the patient what they understood first. Future curricula should use a similar framework with challenging conversations, such as oncology, palliative care, and critical care. There may also be some value in introducing the card to first-year neurology residents during orientation because not all medical schools may offer this type of training.

The implementation of the Communication Card significantly affected patient care in multiple ways. First, it offered an opportunity for more in-depth and private conversations between students and patients outside of rounds. Sometimes, rounds can be rushed due to time constraints, and having additional time with patients could be beneficial for them. The Communication Card addresses this gap, providing a second chance for patients to ask any questions that were missed during rounds. In addition, some patients feel intimidated and overwhelmed because of the nature of rounds, and circling back in a one-on-one more private setting provides an opportunity to ask questions that patients were hesitant to ask in front of many people. Therefore, students perceived that their patients felt more comfortable and had a better understanding of their condition, which could lead to more adherence to treatment, although that needs to be followed longitudinally. In addition, students described their patient's emotional response through further discussions that were started using the Communication Card. Patient's mental states and concerns were not always known to the team before their conversation. These fears may never be voiced if students or other team members do not reapproach patients. With the Communication Card, patients were able to feel more comfortable and trusting of the team because they felt that the team cared for them. Future conversation frameworks should provide additional questions that gather insight into patients' emotions on their condition, rather than just assessing comprehension and understanding. However, our results only show students' perspectives, and further insight into patients' experiences need to be addressed. Including an extension of the Communication Cards to be filled out by patients, where they can express their perspective, could help corroborate whether their satisfaction and understanding was improved or not.

Our study has several limitations. First, the study was retrospectively performed and included a large variety of neurology clerkship sites. With the large variety of sites, there was also a large variety in the student experience at these sites. Some clerkship sites were added or removed throughout the course of the data collection period. Having consistent clerkship sites could form a more robust analysis. Some students mostly had an inpatient experience, with patients who have more comorbidities and subsequently participated in more difficult conversations. Whereas for students who participated in outpatient visits, the patient population at those sites are generally healthier; therefore, students are less likely to participate in difficult conversations at those sites. Second, the free-text format of the Communication Card limited the space available for the student to write their responses. Students were only able to respond to each open-ended question with 2–3 sentences, limiting the length and detail of their responses. This data collection strategy may have affected conceptual richness; future work should explore this topic using more robust methods of data collection.^14^ However, we analyzed each response for its content and appropriately placed them in at least 1 response code in our study to generate an overall understanding of students' experiences. In addition, student and patient characteristics, such as prior communication training, race, ethnicity, gender, and possible language barriers, were not measured. These characteristics could have affected the quality of the conversation between patients and medical students. Another limitation is that because completion of the communication card was a course requirement and that the cards were not anonymous to course directors, this could have affected what students disclosed. In addition, our results are from the perspective of the medical student and may not reflect the patient's reality. Further studies could examine the patient perspective after these conversations and determine whether they corroborate the student's perception.

Overall, we found that the introduction of the Communication Card was an effective teaching tool for medical students during their neurology clerkship. Medical educators should consider implementing a Communication Card or an equivalent tool as a basis for skills training throughout the neurology clerkship, as well as within the preclinical stages of medical training, possibly via standardized patients, to have a more robust curriculum throughout medical school. Our study offers a further step in identifying potential outcomes to communication skills training in medical students during their neurology clerkship.

## Appendix Authors

**Table:**

Name	Location	Contribution
Carmen Priego-Pérez, MD	Facultad de Medicina, Universidad de Córdoba, Spain	Drafting/revision of the article for content, including medical writing for content; study concept or design; and analysis or interpretation of data
Punthitra Arpornsuksant, MD	Department of Neurology, Johns Hopkins Medicine, Baltimore, MD	Drafting/revision of the article for content, including medical writing for content; study concept or design; and analysis or interpretation of data
Rachel Marie E. Salas, MD, MEd	Department of Neurology, Johns Hopkins Medicine, Baltimore, MD	Drafting/revision of the article for content, including medical writing for content; major role in the acquisition of data; and study concept or design
Charlene E. Gamaldo, MD	Department of Neurology, Johns Hopkins Medicine, Baltimore, MD	Study concept or design
Monica Lemmon, MD	Department of Pediatrics and Population Health Sciences, Duke University School of Medicine, Durham, NC	Drafting/revision of the article for content, including medical writing for content
Roy E. Strowd III, MD, MEd, MS	Department of Neurology, Wake Forest University School of Medicine, Winston-Salem, NC	Drafting/revision of the article for content, including medical writing for content
Doris G. Leung, MD, PhD	Department of Neurology, Johns Hopkins Medicine; Kennedy Krieger Institute, Baltimore, MD	Drafting/revision of the article for content, including medical writing for content; major role in the acquisition of data; and study concept or design

## Glossary

COVID-19: coronavirus disease 2019

## References

[1] Lemmon ME, Gamaldo C, Salas RME, et al. Education research: difficult conversations in neurology: lessons learned from medical students. Neurology. 2018;90(2):93-97. doi:10.1212/WNL.0000000000004794 29311368

[2] Warm EJ, Kinnear B, Lance S, Schauer DP, Brenner J. What behaviors define a good physician? Assessing and communicating about noncognitive skills. Acad Med. 2022;97(2):193-199. doi:10.1097/ACM.0000000000004215 34166233

[3] Willis R, Strowd RE, Barks MC, Salas RE, Gamaldo CE, Lemmon ME. Education research: the medical student perspective on challenging conversations. Neurology. 2020;95(5):226-230. doi:10.1212/WNL.0000000000009261 32273429 PMC7455339

[4] Lunn AM, Urmston A, Seymour S, Manfrin A. Patient as teacher sessions contextualize learning, enhancing knowledge, communication, and participation of pharmacy students in the United Kingdom. J Educ Eval Health Prof. 2020;17:15. doi:10.3352/jeehp.2020.17.15 32429014 PMC7344118

[5] Jha V, Quinton ND, Bekker HL, Roberts TE. Strategies and interventions for the involvement of real patients in medical education: a systematic review. Med Educ. 2009;43(1):10-20. doi:10.1111/j.1365-2923.2008.03244.x 19140994

[6] Ogur B, Hirsh D, Krupat E, Bor D. The Harvard Medical School-Cambridge Integrated Clerkship: an innovative model of clinical education. Acad Med. 2007;82(4):397-404. doi:10.1097/ACM.0b013e31803338f0 17414198

[7] Vermylen JH, Wayne DB, Cohen ER, Mcgaghie WC, Wood GJ. Promoting readiness for residency: embedding simulation-based mastery learning for breaking bad news into the medicine subinternship. Acad Med. 2020;95(7):1050-1056. doi:10.1097/ACM.0000000000003210 32576763

[8] Vargovich AM, Schumann ME, Xiang J, Ginsberg AD, Palmer BA, Sperry JA. Difficult conversations: training medical students to assess, educate, and treat the patient with chronic pain. Acad Psychiatry. 2019;43(5):494-498. doi:10.1007/s40596-019-01072-4 31168741

[9] Przymuszała P, Marciniak-Stępak P, Cerbin-Koczorowska M, et al. ‘Difficult conversations with patients’: a modified group objective structured clinical experience for medical students. Int J Environ Res Public Health. 2021;18(11):5772. doi:10.3390/ijerph18115772. 34072179 PMC8197999

[10] Costello J, Horne M. Patients as teachers? An evaluative study of patients' involvement in classroom teaching. Nurse Educ Pract. 2001;1(2):94-102. doi:10.1054/nepr.2001.0014 19036250

[11] Wykurz G, Kelly D. Developing the role of patients as teachers: literature review. BMJ. 2002;325(7368):818-821. doi:10.1136/bmj.325.7368.818 12376445 PMC128951

[12] Miles M, Huberman AM. Qualitative Data Analysis: An Expanded Sourcebook. 2nd ed. SAGE Publications; 1994.

[13] Hsieh HF, Shannon SE. Three approaches to qualitative content analysis. Qual Health Res. 2005;15(9):1277-1288. doi:10.1177/1049732305276687 16204405

[14] LaDonna KA, Taylor T, Lingard L. Why open-ended survey questions are unlikely to support rigorous qualitative insights. Acad Med. 2018;93(3):347-349. doi:10.1097/ACM.0000000000002088 29215376

